# Right and left ventricular function and flow quantification in pediatric patients with repaired tetralogy of Fallot using four-dimensional flow magnetic resonance imaging

**DOI:** 10.1186/s12880-021-00693-2

**Published:** 2021-10-31

**Authors:** Xiaofen Yao, Liwei Hu, Yafeng Peng, Fei Feng, Rongzhen Ouyang, Weihui Xie, Qian Wang, Aimin Sun, Yumin Zhong

**Affiliations:** 1 Department of Radiology, Shanghai Children’s Medical Center, School of Medicine, Shanghai Jiao Tong University, No. 1678 Dongfang Road, Shanghai, 200127 China; 2 AI Imaging, GE Healthcare, No. 1 Huatuo Road, Shanghai, 201203 China

**Keywords:** 4D flow, Cardiac magnetic resonance, Repaired tetralogy of Fallot

## Abstract

**Background:**

To assess the accuracy and reproducibility of right ventricular (RV) and left ventricular (LV) function and flow measurements in children with repaired tetralogy of Fallot (rTOF) using four-dimensional (4D) flow, compared with conventional two-dimensional (2D) magnetic resonance imaging (MRI) sequences.

**Methods:**

Thirty pediatric patients with rTOF were retrospectively enrolled to undergo 2D balanced steady-state free precession cine (2D b-SSFP cine), 2D phase contrast (PC), and 4D flow cardiac MRI. LV and RV volumes and flow in the ascending aorta (AAO) and main pulmonary artery (MPA) were quantified. Pearson’s or Spearman’s correlation tests, paired t-tests, the Wilcoxon signed-rank test, Bland–Altman analysis, and intraclass correlation coefficients (ICC) were performed.

**Results:**

The 4D flow scan time was shorter compared with 2D sequences (P < 0.001). The biventricular volumes between 4D flow and 2D b-SSFP cine had no significant differences (P > 0.05), and showed strong correlations (r > 0.90, P < 0.001) and good consistency. The flow measurements of the AAO and MPA between 4D flow and 2D PC showed moderate to good correlations (r > 0.60, P < 0.001). There was good internal consistency in cardiac output. There was good intraobserver and interobserver biventricular function agreement (ICC > 0.85).

**Conclusions:**

RV and LV function and flow quantification in pediatric patients with rTOF using 4D flow MRI can be measured accurately and reproducibly compared to those with conventional 2D sequences.

## Background

Congenital heart disease (CHD) is the most common birth defect in China [1]. Tetralogy of Fallot (TOF) is the most common cyanotic CHD [2, 3]. Patients with repaired TOF (rTOF) develop pulmonary valve regurgitation, which leads to right ventricular (RV) enlargement and dysfunction. Therefore, ventricular function and flow information, especially RV function and pulmonary regurgitation (PR), must be provided by magnetic resonance imaging (MRI) during follow up of rTOF [4].

Cardiac function and flow measurements by cardiac magnetic resonance (CMR) are usually analyzed using conventional two-dimensional (2D) balanced steady-state free precession (b-SSFP) cine and 2D phase contrast (PC) sequences. The accuracy of 2D b-SSFP cine has been validated and the technique is widely used in postoperative functional assessment with CHD [5–10]. 2D PC is the primary method used to measure blood flow volume and velocity in CMR [11]. However, the conventional 2D b-SSFP cine and 2D PC sequences are relatively time-consuming. Each sequence highly depends on the technologist to determine appropriate scan planes and parameters, which limits its availability [12, 13]. Since long acquisition time and extended sedation make CMR difficult for young children, four-dimensional (4D) flow can assess both ventricular function and flow information with only one sequence, the imaging time can be significantly saved [14, 15].

In this study, we aimed to assess the RV and left ventricular (LV) function and flow measurements in children patients with rTOF using 4D flow, compared with conventional 2D MRI sequences.

## Methods

### Patient population

We retrospectively identified pediatric patients with rTOF who were referred for CMR at our hospital and informed consent was waived. We included patients with rTOF who underwent 2D CMR (2D cine b-SSFP, 2D PC) and 4D flow MRI for ventricular function and flow assessment from September 2018 to September 2019.

### Image acquisition

All images were performed on a 3.0-T MRI scanner with an eight-channel phased-array cardiac coil (Discovery 750, GE Healthcare, Waukesha, WI, USA) with electrocardiography (ECG) gating. Deep sedation was used in patients aged under 6 years.

Conventional 2D b-SSFP cine planes were acquired in a four-chamber view, two- and three-chamber views, and a short axis view. Then, 2D PC and whole-heart 4D flow sequences were performed after administration of gadolinium contrast agent (0.05–0.10 mmol/kg injected intravenously at 1.0–1.5 ml/s, Magnevist, Bayer). Velocity encoding (VENC) for 2D PC was 150–200 cm/s for the ascending aorta (AAO) and 150–380 cm/s for the main pulmonary artery (MPA) according to the results of the recent echo.

4D flow acquisition was performed in coronal or axial plane full volumetric coverage of the great arteries with ECG gating (30 interpolated phases per cardiac cycle) and without respiratory triggering. With the acceleration technique of kt-ARC and the parallel imaging with reduction factor R of 2, the resulting scan time was on the order of 5–10 min. We selected thinner slice thickness in order to achieve isotropic voxels [16, 17]. The range of VENC for 4D flow was 120–380 cm/s in all three directions according to the results of echo as 2D PC. The acquisition parameters expressed as a range of these three sequences were detailed in Table 1.

**Table 1:** The acquisition parameters in 2D b-SSFP cine, 2D PC and 4D flow sequences

	2D b-SSFP cine	2D PC	4D flow
Repetition time (TR, ms)	3.46 (3.22–3.73)	5.56 (4.97–6.03)	4.58 (4.31–5.04)
Echo time (TE, ms)	1.54 (1.43–1.66)	2.95 (2.38–3.16)	2.22 (2.09–2.40)
Flip angle (°)	45/50	20	8–15
Views per Segment	12–14	2–4	/
Temporal resolution (ms)	55.36 (51.52–59.68)	44.48 (39.76–48.24)	36.63 (34.48–40.32)
Acquired spatial resolution (mm)	1.5–2.5 × 1.5–2.5	1.5–2.5 × 1.5–2.5	1.02–2.00 × 1.02–2.00
Slice thickness(mm)	5.0–8.0	4.0–5.0	1.00–2.00
Interpolated Cardiac phases/cycle	30	30	30
VENC (cm/s)	N/A	150–380	120–380
Navigator gating	No	No	No
ECG triggering	Yes	Yes	Yes

### Image postprocessing

**Fig. 1:**
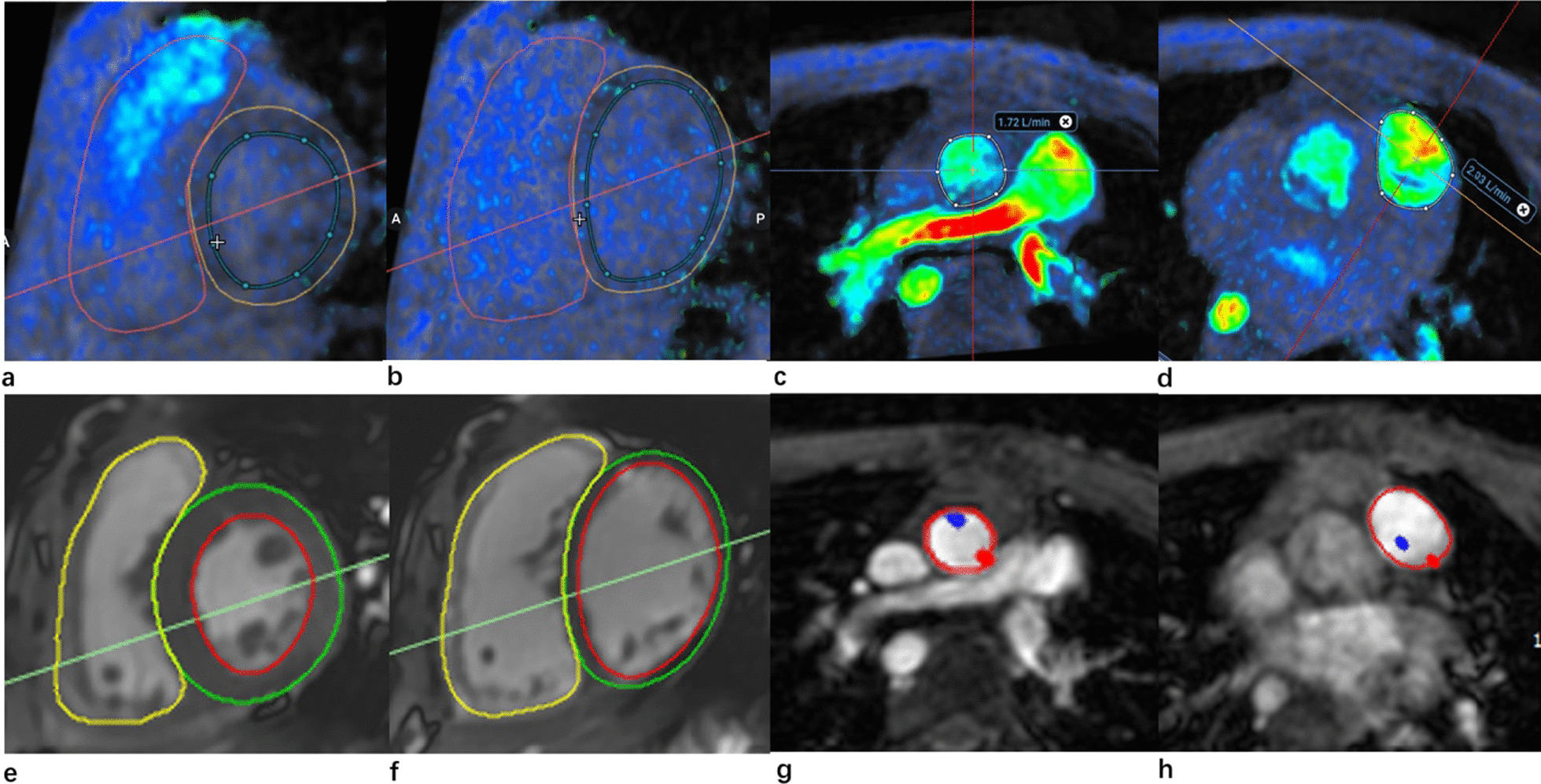
Ventricular function and flow quantification measured by 4D flow, 2D b-SSFP cine and 2D PC for a 5-year-old male patient with rTOF. 4D flow short axis screen captures of LV and RV end-systole (**a**) and end-diastole (**b**), with through plane 4D flow images of the AAO (**c**) and MPA (**d**), and 2D b-SSFP cine short axis screen captures of LV and RV end-systole (**e**) and end-diastole (**f**), with through plane 2D PC images of the AAO (**g**) and MPA (**h**). Reformatted 4D flow images on the upper row are overlaid with color velocity to identify the myocardial blood boundary

Automated corrections were preprocessed on 2D PC or 4D flow to avoid aliasing artefacts. To acquire cardiac volume results using 4D flow using dedicated software (Arterys Inc., San Francisco, CA, USA), short-axis and axial imaging planes were automatically generated. After manual segmentation of the LV and RV (Fig. 1), end-diastolic volume indexed (EDVi), end-systolic volume indexed (ESVi), stroke volume indexed (SVi), ejection fraction (EF), and cardiac output indexed (COi) were computed at end-diastole and end-systole. All ventricular volume and function measurements were normalized to body surface area (BSA) using the Mosteller method.

To quantify AAO and MPA flow with 4D flow using the Arterys platform, AAO and MPA cross-sectional planes were reconstructed perpendicular to the direction of flow in early systole. AAO flow was measured at the midpoint of the AAO. Because there were no clearly defined pulmonary valves in patients with rTOF, MPA flow was measured at the midpoint of the pulmonary trunk. The AAO and MPA contours were adjusted at different time points, and then the net flow, forward flow, peak velocity, and regurgitation fraction (RF) of both AAO and MPA were calculated automatically (Fig. 1). The AAO and MPA flow volumes were multiplied by the related forward flow and heart rate values.

2D b-SSFP cine and 2D PC analyses were performed using Circle Cardiovascular Imaging software (CVI42 v.5.9.3, Circle Cardiovascular Imaging Inc., Calgary, Canada). Each sequence was manually processed by the board-certified radiologist with specialty training in pediatric cardiac imaging, which was the same person who postprocessed the 4D flow sequence (Fig. 1). Trabeculations and papillary muscles of the LV and RV were included as part of the ventricular cavity and a smooth endocardial border was drawn to improve reproducibility [18]. After segmenting 2D PC sequences, net flow, forward flow, peak velocity, and RF in both the AAO and MPA were obtained.

### Statistical analysis

Statistical analyses were performed using SPSS 25.0 software (Chicago, IL, USA) and GraphPad Prism 7 (San Diego, CA, USA), and a significance level of 0.05 was applied for all statistical tests. Continuous variables were checked for a normal distribution using the Shapiro–Wilk test, and expressed as mean ± standard deviation (SD). Categorical variables are presented as number (%). Either Pearson’s or Spearman’s correlation test was used to test the correlation in the results between 4D flow and 2D MR sequences according to the variable distribution. Correlation (r) was considered poor for values between 0.30 and 0.50, moderate for values between 0.50 and 0.70, and good for values between 0.70 and 1.00. A paired t-test or the Wilcoxon signed-rank test was used to test the difference between 4D flow and 2D sequences. The agreement in measurements between 4D flow and 2D MR sequences was assessed by Bland–Altman analysis, which calculated the mean difference or mean percentage difference between measurements and 95% limits of agreement (LOA, mean ± 1.96 SD). The intraclass correlation coefficients (ICC) with 95% confidence interval (CI) were applied to test intraobserver and interobserver reproducibility between 4D flow and 2D b-SSFP cine. Interobserver reproducibility in ventricular function was assessed by two radiologists both with > 3 years’ experience in reading CMR in a double-blinded manner.

## Results

### Demographics

Demographic information of all 30 patients with rTOF underwent CMR were summarized in Table 2. There was no significant difference (P = 0.724) in heart rate between 4D flow (77.03 ± 13.30 bpm) and 2D acquisition (76.60 ± 12.38 bpm). The scan times of 4D flow and 2D sequences were 8.10 ± 2.25 min and 34.66 ± 7.41 min, respectively (P < 0.001). The postprocessing times of 4D flow and 2D sequences were about 40 min and 45 min respectively. The echocardiography performed at the same time as CMR did reported that 21 patients had mild tricuspid regurgitation, one patient had the residual interventricular shunt and eight patients had interatrial shunt. Three cases were experienced minor aliasing on 4D flow and were corrected automatically by Arterys.

**Table 2:** Summary of patient demographics

	All patients (n = 30)
Male (%)	80
Height (cm)	119.97 ± 28.52
Weight (kg)	23.83 ± 14.57
BSA (m^2^)	0.89 ± 0.37
Age at CMR (years)	6.30 ± 4.19
Age at TOF repair (months)	10.88 ± 7.01
Duration between surgery and CMR (years)	5.62 ± 4.06

### Comparison of ventricular function

**Fig. 2:**
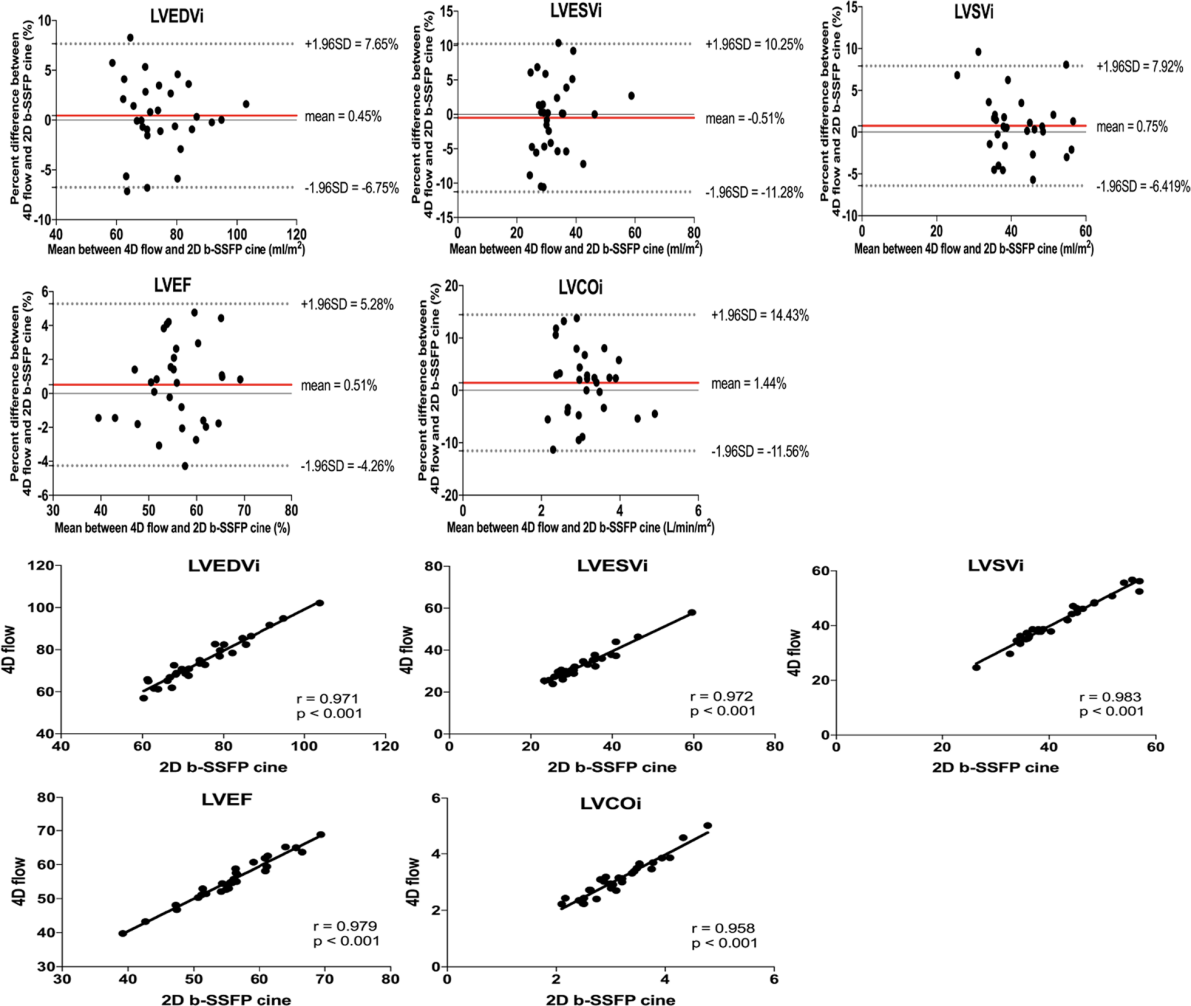
Bland–Altman and correlation plots in LVEDVi, LVESVi, LVSVi, LVEF, and LVCOi between 4D flow and 2D b-SSFP cine. The mean percentage differences and LOA are represented with red and gray dashed lines, respectively

**Fig. 3:**
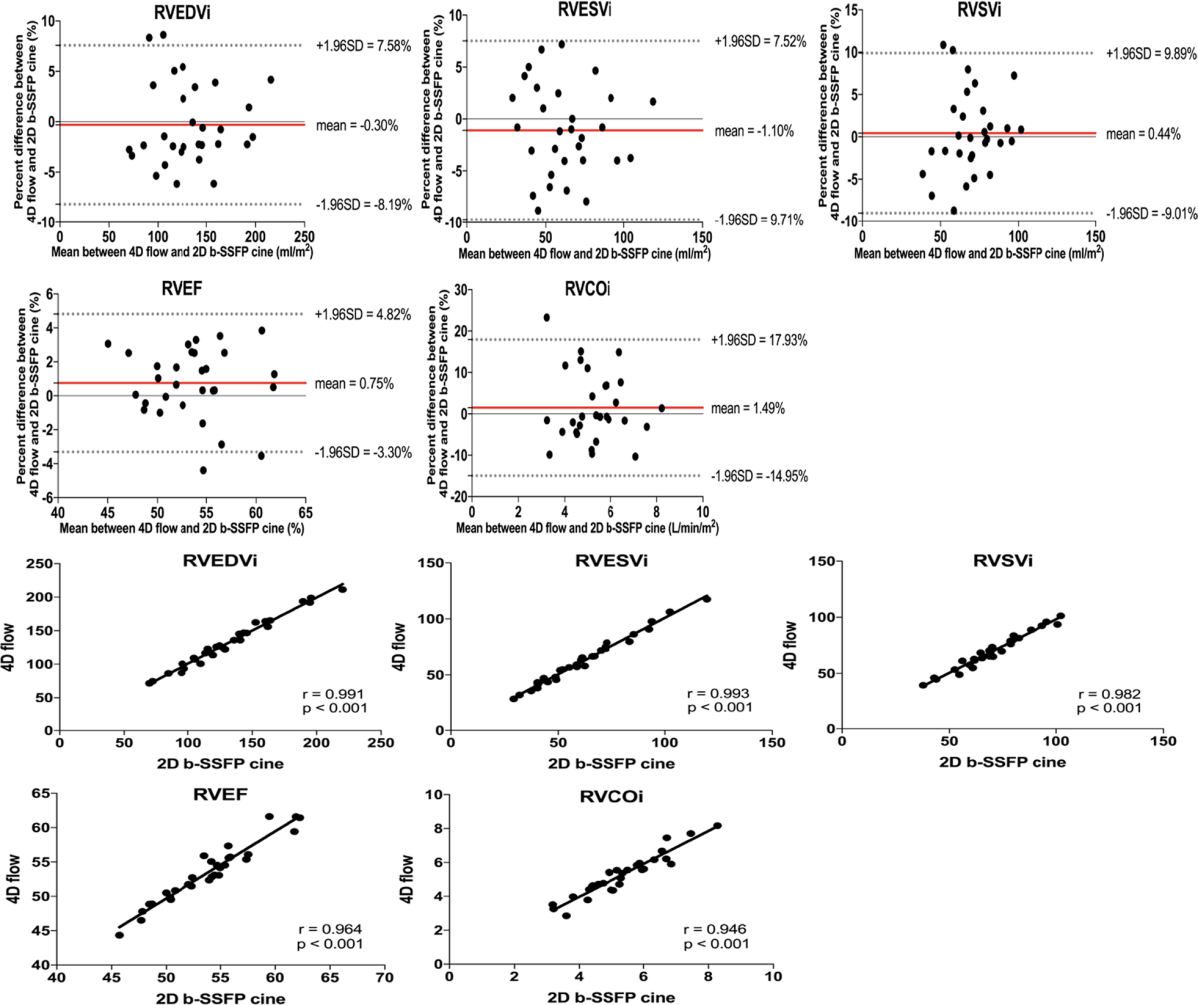
Bland–Altman and correlation plots in RVEDVi, RVESVi, RVSVi, RVEF, and RVCOi between 4D flow and 2D b-SSFP cine. The mean percentage differences and LOA are represented with red and gray dashed lines, respectively

The ventricular function of both ventricles was shown in Table 3 for 2D cine b-SSFP and 4D flow short axis view. The correlations in biventricular function between CMR 4D flow and 2D b-SSFP cine sequences were strong (r > 0.90, P < 0.001; Table 3). There were no significant differences (P > 0.050) in biventricular function between 4D flow and 2D b-SSFP cine sequences. As Figs. 2 and 3 showed, the mean percentage differences in biventricular function between 4D flow and 2D b-SSFP cine were near zero, and the biventricular LOA were narrow and the RV volume LOA between 4D flow and b-SSFP were slightly wider when compared with the LV volume LOA.

**Table 3:** Comparison of Ventricular Function Data between 2D b-SSFP cine and 4D Flow

Measurements	4D flow short axis view (mean ± SD)	2D b-SSFP cine (mean ± SD)	Paired t-test/Wilcoxon	Correlation	
			P-value	r-value	P-value
LVEDVi (ml/m^2^)	74.23 ± 10.66	74.54 ± 10.60	0.509	0.971	< 0.001
LVESVi (ml/m^2^)	32.75 ± 7.06	32.66 ± 7.47	0.888	0.972	< 0.001
LVSVi (ml/m^2^)	41.48 ± 7.98	41.74 ± 7.86	0.347	0.983	< 0.001
LVEF (%)	55.81 ± 6.70	56.11 ± 6.87	0.238	0.979	< 0.001
LVCOi (L/min/m^2^)	3.10 ± 0.67	3.14 ± 0.63	0.455	0.958	< 0.001
RVEDVi (ml/m^2^)	132.77 ± 36.68	132.40 ± 36.85	0.691	0.991	< 0.001
RVESVi (ml/m^2^)	62.87 ± 21.95	62.11 ± 21.67	0.124	0.993	< 0.001
RVSVi (ml/m^2^)	69.90 ± 15.78	70.31 ± 16.26	0.471	0.982	< 0.001
RVEF (%)	53.43 ± 4.31	53.83 ± 4.25	0.067	0.964	< 0.001
RVCOi (L/min/m^2^)	5.25 ± 1.25	5.32 ± 1.22	0.371	0.946	< 0.001

The right ventricular function using the 4D flow axial plane and the results were shown in Tables 4 and 5. There were no significant differences (P > 0.050) when comparing the right ventricular volume measurements in 4D flow axial plane to those of 2D b-SSFP cine or those of 4D short axis view. Besides, the correlations of right ventricular function were strong (r > 0.85, P < 0.001). And the mean differences in right ventricular volumes were near zero and the LOA were narrow whether comparing the right ventricular volume measurements in 4D flow axial plane to those of 2D b-SSFP cine or those of 4D short axis view.

**Table 4:** Comparison of right ventricular function data between 2D b-SSFP cine and 4D flow axial plane

Measurements	Paired t-test/Wilcoxon	Correlation		Bland–Altman	
	P-value	r-value	P-value	LOA	Mean difference
RVEDVi (ml/m^2^)	0.671	0.999	< 0.001	(− 2.16, 2.24)	0.04 ± 1.12
RVESVi (ml/m^2^)	0.652	0.997	< 0.001	(− 5.78, 5.17)	− 0.30 ± 2.80
RVSVi (ml/m^2^)	0.467	0.993	< 0.001	(− 4.88, 5.70)	0.41 ± 2.70
RVEF (%)	0.388	0.958	< 0.001	(− 4.08, 4.85)	0.39 ± 2.28
RVCOi (L/min/m^2^)	0.634	0.891	< 0.001	(− 19.94, 18.64)	− 0.65 ± 9.84

**Table 5:** Comparison of right ventricular function data between 4D flow short axis view and 4D flow axial plane

Measurements	Paired t-test/Wilcoxon	Correlation		Bland–Altman	
	P-value	r-value	P-value	LOA	Mean difference
RVEDVi (ml/m^2^)	0.470	0.998	< 0.001	(− 4.11, 3.51)	− 0.30 ± 1.94
RVESVi (ml/m^2^)	0.764	0.997	< 0.001	(− 6.93, 7.01)	− 0.04 ± 3.56
RVSVi (ml/m^2^)	0.326	0.992	< 0.001	(− 6.30, 5.26)	− 0.52 ± 2.95
RVEF (%)	0.568	0.957	< 0.001	(− 4.81, 4.37)	− 0.22 ± 2.34
RVCOi (L/min/m^2^)	0.158	0.979	< 0.001	(− 13.07, 9.34)	− 1.87 ± 5.72

### Comparison of AAO and MPA flow quantification

We compared the consistency of net flow, forward flow, peak velocity, and RF of the AAO and MPA between 4D flow and 2D PC, which showed moderate to good correlation and agreement (Table 6). The AAO and MPA correlations of net flow, forward flow, peak velocity, and RF were moderate to good (r = 0.643–0.923, P < 0.001), and the peak velocity showed the weakest correlation among the three flow measurements for the AAO and the MPA. There was a wider LOA for MPA flow quantification compared with AAO between 4D flow and 2D PC, and the mean differences in MPA flow measurements were larger when compared with AAO between 4D flow and 2D PC.

**Table 6:** 4D flow and 2D PC correlation and agreement for great vessels flow quantification

	4D flow (mean ± SD)	2D PC (mean ± SD)	Correlation	LOA	Mean Difference
AAO net flow (ml/beat)	29.14 ± 12.10	32.06 ± 12.26	0.816	(− 6.81, 12.64)	2.92 ± 4.96
AAO forward flow (ml/beat)	29.67 ± 12.41	33.12 ± 12.99	0.923	(− 6.40, 13.31)	3.45 ± 5.03
AAO peak velocity (cm/s)	83.57 ± 14.32	77.59 ± 11.27	0.643	(− 25.58, 13.63)	− 5.98 ± 10.00
AAO RF (%)	1.70 ± 1.42	2.91 ± 2.19	0.752	(− 1.63, 4.05)	1.21 ± 1.45
MPA net flow (ml/beat)	36.90 ± 18.00	33.21 ± 14.93	0.831	(− 21.50, 14,13)	− 3.69 ± 9.09
MPA forward flow (ml/beat)	55.48 ± 30.12	60.12 ± 29.09	0.891	(− 22.51, 31.78)	4.64 ± 13.85
MPA peak velocity (cm/s)	159.13 ± 50.34	151.92 ± 53.53	0.779	(− 75.12, 60.70)	− 7.21 ± 34.65
MPA RF (%)	29.93 ± 13.85	41.75 ± 13.62	0.790	(− 3.91, 27.54)	11.82 ± 8.02

### Comparison of internal consistency with 4D flow and 2D sequences

**Fig. 4:**
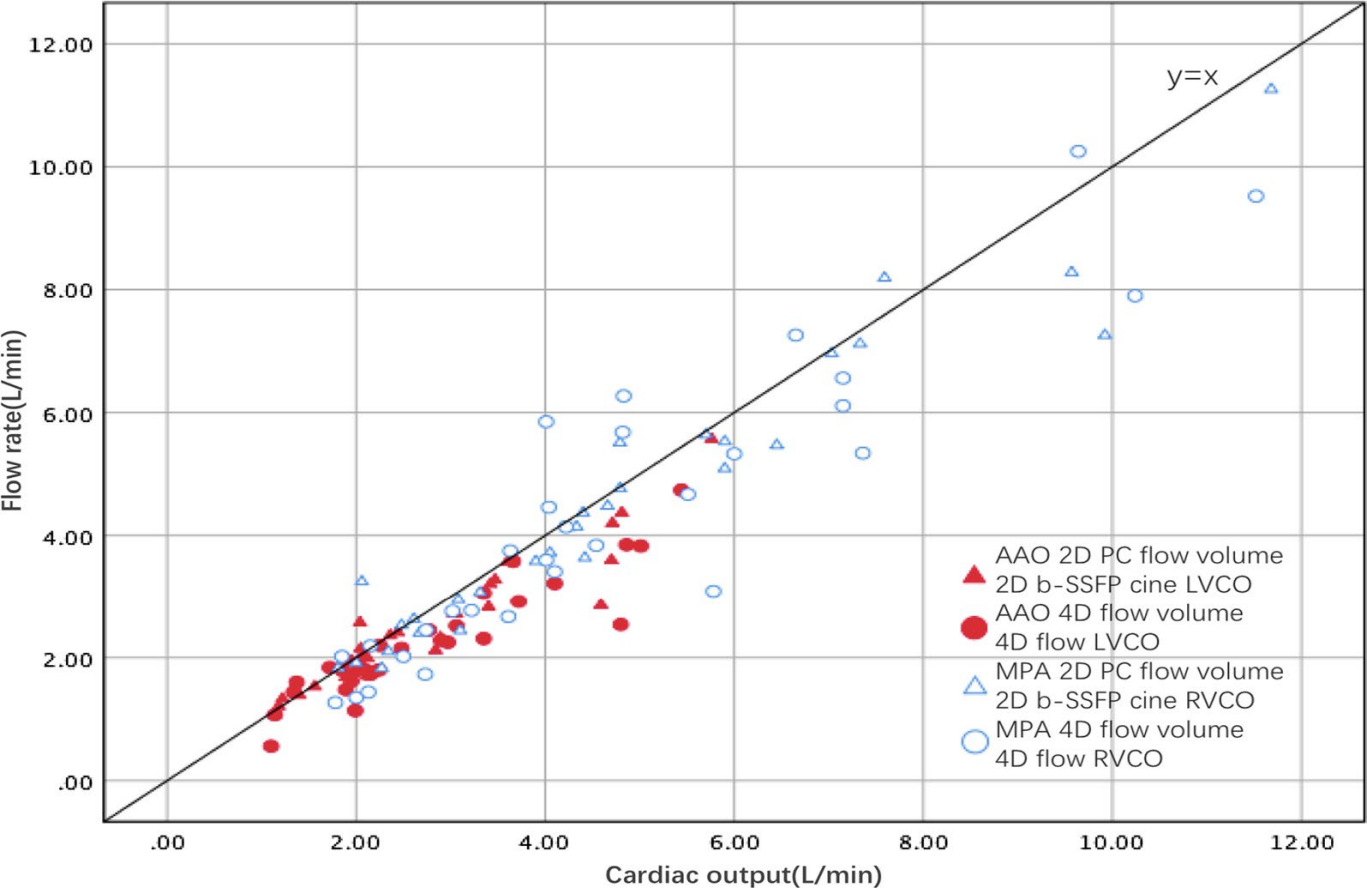
Comparison of cardiac output between the systolic forward flow volumes (y-axis) estimated by 4D flow or 2D PC and ventricular volumes (x-axis) estimated by 4D flow or 2D b-SSFP cine. Systemic measurements are displayed in red closed symbols and pulmonary measurements in blue open symbols, while measurements by 2D PC or 2D b-SSFP cine are displayed in triangles and 4D flow measurements in circles. Scatter plots show the correlation between the 4D or 2D forward flow volumes and 4D or 2D ventricular volumes of the LV or RV

We also applied internal consistency for systemic and pulmonary flow volumes between 4D flow and 2D sequences by comparing CO estimated from LVSV or RVSV and CO estimated from AAO or MPA forward volumes (Table 7, Fig. 4). The correlations in LVCO or RVCO and AAO or MPA forward volumes measured by 4D flow and 2D sequences were good (r > 0.80, P < 0.001). There was a narrow LOA between LVCO or RVCO and AAO or MPA forward flow volume with both 4D flow and 2D PC, and the mean differences were near zero for both 4D flow and 2D sequences. With a minor mean aortic RF (< 10%) (Table 6) in patients with rTOF, the red closed symbols in the scatter plot (Fig. 4) were near the line of identity (y = x), which validated that LVCO was well matched to AAO forward flow volume with 2D PC and 4D flow. Despite the mean pulmonary RF was moderate to severe (> 20%) (Table 6), the blue open symbols in the scatter plot (Fig. 4) were near the line of identity (y = x), which validated that RVCO was well matched to MPA flow forward volume with 2D PC and 4D flow.

**Table 7:** Correlation and agreement of LVCO and AAO forward flow volume between 4D flow and 2D sequences

	Correlation	LOA	Mean difference
4D LVCO vs. 4D AAO forward flow volume	0.924	(− 1.52, 0.47)	− 0.52 ± 0.51
2D LVCO vs. 2D AAO forward flow volume	0.944	(− 1.21, 0.54)	− 0.34 ± 0.45
4D RVCO vs. 4D MPA forward flow volume	0.834	(− 2.20, 3.33)	0.57 ± 1.41
2D RVCO vs. 2D MPA forward flow volume	0.967	(− 1.01, 1.56)	0.27 ± 0.66

### Intraobserver and interobserver reproducibility for ventricular function

The intraobserver and interobserver ICCs of biventricular function for both 4D flow and 2D b-SSFP cine were summarized in Table 8. Intraobserver and interobserver reproducibility demonstrated very well (ICC > 0.85) with both 4D flow and 2D b-SSFP cine when quantifying LV and RV function.

**Table 8:** Intraobserver and interobserver reproducibility for quantifying ventricular volumes

	Intraobserver reproducibility (n = 10)		Interobserver reproducibility (n = 10)	
	4D flow ICC (95%CI)	2D b-SSFP cine ICC (95%CI)	4D flow ICC (95%CI)	2D b-SSFP cine ICC (95%CI)
LVEDVi	0.992 (0.971, 0.998)	0.996 (0.985, 0.999)	0.991 (0.965, 0.997)	0.995 (0.982, 0.999)
LVESVi	0.997 (0.940, 0.999)	0.999 (0.989, 0.999)	0.995 (0.981, 0.991)	0.997 (0.990, 0.999)
LVSVi	0.989 (0.960, 0.997)	0.998 (0.991, 0.999)	0.977 (0.915, 0.994)	0.988 (0.956, 0.997)
LVEF	0.979 (0.925, 0.994)	0.990 (0.961, 0.997)	0.951 (0.828, 0.986)	0.975 (0.906, 0.993)
LVCOi	0.947 (0.817, 0.985)	0.973 (0.899, 0.993)	0.906 (0.690, 0.974)	0.939 (0.789, 0.983)
RVEDVi	0.949 (0.809, 0.986)	0.977 (0.914, 0.994)	0.902 (0.679, 0.973)	0.955 (0.842, 0.988)
RVESVi	0.953 (0.826, 0.987)	0.986 (0.948, 0.996)	0.911 (0.704, 0.975)	0.973 (0.902, 0.993)
RVSVi	0.978 (0.917, 0.994)	0.971 (0.893, 0.992)	0.956 (0.847, 0.988)	0.944 (0.806, 0.985)
RVEF	0.972 (0.895, 0.992)	0.973 (0.898, 0.993)	0.945 (0.809, 0.985)	0.947 (0.815, 0.985)
RVCOi	0.951 (0.816, 0.987)	0.968 (0.882, 0.991)	0.861 (0.565, 0.961)	0.925 (0.722, 0.980)

## Discussion

CMR is the reference method used to assess ventricular volume, function, and flow as well as longitudinal follow up of patients over time [8, 19, 20]. 2D b-SSFP cine is widely preferred for the evaluation of cardiac function with lower interobserver variability and good blood myocardium contrast [21, 22]. 2D PC is the primary method used to quantify blood flow with the magnitude image used to provide anatomical information and the phase image used to provide velocity information [11, 23]. All 2D multiplanar sequences require a relatively long scan time, which is challenging for pediatric patients who cannot do MRI or who require deep sedation [24]. Previous studies have demonstrated that 4D flow MRI can provide flow information and assess cardiac function precisely and reliably [14, 25, 26]. Our study confirmed these findings in pediatric patients with rTOF.

The 4D flow results of biventricular function compared with 2D b-SSFP cine sequences demonstrated a more accurate ventricular volume assessment compared with prior published research [14, 25, 26]. A previous study showed that contrast agent has been validated to improve the signal-to-noise ratio and suppress background noise [27]. Our ventricular results indicated that 4D flow with contrast agent could provide an adequate image quality to acquire precise ventricular function measurements comparable to 2D b-SSFP cine. However, RV LOA of volumes between 4D flow and b-SSFP were relatively wider when compared with LV LOA of volumes in this study, which was attributable to the irregular and enlarged RV geometry in patients with rTOF especially in basal slices’ segmentation, or because the relatively thinner RV myocardial wall made it harder to delineate than the LV. With the advantage of 4D flow 3D dataset, different from previous studies [14, 25, 26], both the short axis plane and axial plane could be obtained during the post-processing of 4D flow, and could provide reliable measurements for follow-up of the right ventricular function in patients with rTOF.

The net flow, forward flow and RF of the AAO and MPA measured by 4D flow and 2D PC demonstrated a moderate to good correlation (r > 0.60, P < 0.001) and agreement. Nevertheless, peak velocity and RF measurements showed relatively poorer correlation and agreement compared with net flow and forward flow for both AAO and MPA by 4D flow or 2D PC, which was significantly affected by the orientation of the AAO and MPA image plane by 4D flow or 2D PC with no use of valve-tracking, and was also influenced by the turbulent flow in 2D PC and 4D flow [18]. Though the mean difference of RF between 4D flow and 2D PC was around 10%, which is considered clinically acceptable [28]. As the prior study showed, patients with rTOF may have a combination of pulmonary stenosis and regurgitation, which can lead to turbulent flow, dephasing within a volume, and resultant signal loss with PC MRI [4]. Our pulmonary regurgitation was measured at the MPA both by 2D PC and 4D flow, while the prior studies showed that PR measured at the pulmonary valve and valve tracking method can further improve reliability and accuracy of flow measurements [4, 26]. Our study indicated that 4D flow showed a higher peak velocity estimation in both the AAO and MPA compared with 2D PC, which is consistent with a previous study [28]. This study [28] demonstrated that 4D flow could analyze peak velocity with better accuracy than 2D PC compared with the echo standard. The greater mean difference and wider LOA for MPA flow were detected compared with AAO flow measurements between 2D PC and 4D flow. This was due to the complicated hemodynamics in patients with rTOF, which may be caused by pulmonary valve insufficiency and RV enlargement after RV outlet tract correcting surgery [4].

To further test the accuracy of 4D flow measurements, we used internal consistency validation by comparing AAO and MPA forward flow volume obtained by 4D flow or 2D PC with LVCO and RVCO obtained by 4D flow or 2D cine b-SSFP [16]. According to the internal consistency of systemic forward flow volumes between 4D flow and 2D sequences, LVCO was matched to AAO forward flow volumes in all 30 cases with minor aortic RF (< 10%) in both 4D flow and 2D sequences. Besides, RVCO was also matched to the forward volume in the MPA while 29 out of the 30 patients with rTOF had significant (> 10%) MPA regurgitation by 2D PC. The whole exact inlet and outlet match of the RVCO and LVCO showed good internal consistency in ventricular function and flow assessment in both the LV and RV between 4D flow and 2D CMR sequences, which showed that the 4D method had a similar accuracy and addressed the occasional discrepancy to evaluate ventricular function and AAO and MPA flow in postoperative patients with TOF compared with the reference 2D method.

The ICC results demonstrated high intraobserver and interobserver reproducibility (ICC > 0.85) of biventricular function between 4D flow and 2D b-SSFP cine. For both 2D and 4D flow sequences, intraobserver and interobserver ICC of LV measurements having greater agreement than RV measurements, while our results had better RV ICC than previously published values [24]. Due to the abnormal RV geometry in patients with rTOF, it is challenging to quantify RV volume accurately and reliably. Importantly, the intraobserver and interobserver ICCs of 4D flow volume measurements for both the LV and RV were similar to 2D b-SSFP cine. In addition, greater interobserver variability was noted for EF and COi measurements (ICC = 0.861–0.975), which may be explained by the fact that the error in two independent volume measurements may be increased by dividing them. Compared to prior reports [25], volume measurements in 4D flow and 2D b-SSFP cine had equally well intraobserver and interobserver reproducibility.

4D flow with a short scanning time is an ideal technology that provides comprehensive assessment of cardiac function and flow quantification simultaneously for patients with rTOF [4, 29]. Furthermore, 4D flow data are obtained for all parameters in a single identical heart rate and hemodynamic status, while the 2D data are obtained during different time points when the heart rate and hemodynamics change continuously, which can sometimes be significant. What’s more, 4D flow allows more precise prescription of the planes for flow measurement during postprocessing. The measurement plane can be adjusted for each cardiac phase according to the changing direction of flow or the motion of the object structure. Although valve tracking was not employed in this study, prior studies demonstrated that valve tracking method can further improve reliability and accuracy of flow measurements [26, 30]. Lastly, as any vessel included in the imaging volume can be assessed after imaging, 4D flow provides unlimited opportunity for internal validation.

This study had several limitations as below. First, this was a single-center study without inter-institution and inter-software assessment. Second, this study was limited by the small sample involved, which only included patients with post-operative TOF and was not heterogeneous with other types of CHD. Lastly, even quicker 4D flow imaging and compressed sense 2D imaging may be available soon for further studies [31, 32].

## Conclusions

RV and LV function and flow quantification in pediatric patients with rTOF using 4D flow MRI can be measured accurately and reproducibly compared to those with conventional 2D sequences. 4D flow MRI has good potential for clinical application, especially in pediatric patients.

## Data Availability

The datasets used and analyzed during the current study are available from the corresponding author on reasonable request.

## Abbreviations

RV: Right ventricular
LV: Left ventricular
rTOF: Repaired tetralogy of Fallot
4D: Four-dimensional
2D: Two-dimensional
MRI: Magnetic resonance imaging
b-SSFP: Balanced steady-state free precession
PC: Phase contrast
AAO: Ascending aorta
MPA: Main pulmonary artery
ICC: Intraclass correlation coefficients
CHD: Congenital heart disease
PR: Pulmonary regurgitation
CMR: Cardiac magnetic resonance
ECG: Electrocardiography
VENC: Velocity encoding
TR: Repetition time
TE: Echo time
EDVi: End-diastolic volume indexed
ESVi: End-systolic volume indexed
SVi: Stroke volume indexed
EF: Ejection fraction
COi: Cardiac output indexed
BSA: Body surface area
RF: Regurgitation fraction
SD: Standard deviation
LOA: Limits of agreement
CI: Confidence interval
